# The Influence of Social Support and Ability Perception on Coping Strategies for Competitive Stress in Soccer Players: The Mediating Role of Cognitive Assessment

**DOI:** 10.3389/fpsyg.2021.554863

**Published:** 2021-05-25

**Authors:** Zhao Dai, Qiang Liu, Wenhui Ma, Chengwei Yang

**Affiliations:** ^1^College of Physical Education, Sichuan Normal University, Chengdu, China; ^2^Department of Physical Education, Aba Teachers University, Aba, China; ^3^North China Institute of Science and Technology, Langfang, China

**Keywords:** soccer player, social support, ability perception, cognitive assessment, competitive stress, coping strategies

## Abstract

**Objectives:** To explore the effect of social support and ability perception on stress coping strategies for competitive stress, and to reveal the mediating effects of primary and secondary evaluation, so as to further improve the theoretical model of stress coping in soccer players.

**Methods:** A total of 331 male athletes from 22 teams in the Chengdu Middle School Campus Football League were taken as survey samples, and surveys were conducted on their stress experience, social support, ability perception, cognitive assessment, and coping strategies for competition stress. SPSS 21.0 and AMOS 21.0 statistical analysis software were used. Descriptive statistics, correlation analysis, exploratory factor analysis (EFA), and confirmatory factor analysis (CFA) were used to process the obtained data.

**Results:** (1) Positive primary assessment acted as a full mediator in the relationship between social support and secondary assessments, and negative primary assessment acted as a partial mediator in the relationship between competence perception and secondary assessments; (2) Secondary assessment played a partial mediating role between positive primary assessment and positive coping strategies, and a full mediating role between negative primary assessment and positive coping strategies; (3) Secondary assessments played the mediator neither between social support and a coping strategy for stress nor competence perception and a coping strategy for stress; (4) Positive primary assessment, positive negative assessment, and secondary assessment all had significant positive benefits for positive coping. Still, the impact of positive primary assessment on positive coping was significantly better than negative primary assessment and secondary assessment.

**Conclusion:** The coping strategy for the competitive stress model proposed by this study has a very good fit for the causal model. It can be used to explain the observed data from soccer players in middle schools. The primary and secondary assessments play different roles in the model. The combination of problem focuses and emotional focus on the positive stress coping strategy is suitable in the field of competitive sports. Still, the relevant research results need to be further explored and verified in the future.

## Introduction

In recent years, stress coping strategies have become more and more popular with psychologists, athletes, and coaches in the field of athletics, and view them as basic problems that people often face in daily life, study, and work (Nicholls et al., 2016; Hayward et al., 2017). Stress is a physical, mental, or emotional factor that causes bodily or mental tension. Stresses can be external factors from the environment, psychological, or social situations or internal factors from illness, or a medical procedure (Lazarus and Folkman, 1984). Thus, stress has a relationship between individuals and social situations, and if individuals’ assessment on the load imposed on them by this relationship beyond their internal resources will impair their well-being and physical and mental health (Maltby and Day, 2003). The stress response is a complex reaction of urological and endocrinologic system, which can cause continuous cognitive change and behavioral effort. It contains five elements, which are the occurrence of possible stressful events, the primary cognitive assessment of the event, the secondary cognitive assessment of the event, the use of coping styles, and the consequences of physical and mental health. Cognitive assessment is a process of self-assessment. Individuals often continuously interact with cognition, experience, and the external environment to achieve a mental dynamic balance (Delahaij and van Dam, 2017). Thus, cognitive assessment includes primary assessment (i.e., a rapid, initial examination of an individual to recognize and manage all immediate life-threatening conditions), secondary assessment (i.e., a continuation of the primary assessment, where the profession obtains vital signs, reassesses changes in the individual’s conditions, and performs appropriate physical examinations), and individuals will respond according to these assessments. As a result, assessment and response can affect adaptation outcomes, so assessment and response are important variables between stressful individuals and social situations relationships and the immediate and long-term outcomes of such relationships (Kaiseler et al., 2009; Ma et al., 2010; Gourounti et al., 2012; Hayward et al., 2017).

The relationship between cognitive assessment and stress coping strategies has long attracted scholars’ attention (Lazarus and Folkman, 1984; Olmedilla et al., 2018). Alhurani et al. (2018) have found that when the individuals thought that the personal assessment situation can be changed, they tend to use problem-focused coping. If the assessment situation cannot be changed, they use more emotion-focused coping. Thus, the assessment of situational control (secondary assessment) will influence the choice of coping strategies of individuals. Gao et al.’s (2020) research has found that the event-controlled assessment (secondary assessment) in college students will affect their choice of coping strategies, which is manifested by the degree of perceived control of stress events and coping strategies that increase the use of focus. Helbig and Backhaus (2017) conducted a path analysis of stress events and found that the controllable assessment of stress events did not mediate the selection of stress event assessment and coping strategies. Smeds et al. (2019) found that ability perception and social support can simultaneously predict cognitive assessment and coping strategies in stressful situations of general learning situations. Among them, emotional support in social support judges cognitive assessment and coping in the process of personal stress. The choice of coping strategies has great influence. In recent years, the field of competitive sports has also opened up research enthusiasm for stress coping strategies and has obtained a considerable number of research results. Azizi’s (2011) research found that high-level athletes tend to adopt certain coping strategies, such as being proactive, increasing effort, and determination. Goyen and Anshel (1998) explored the approach and avoidance of coping styles of athletes, as well as coping strategies for work and emotions. They found that the responses of athletes are consistent under various stressors. They believe that situational control (secondary assessment) is the intermediary between personal characteristics and situations. In the study of the pre-variable for stress coping strategies, scholars have found that during the process of stress coping, they are affected by different individual differences (such as control beliefs, self-confidence, and ability perception) and environmental factors (Goyen and Anshel, 1998; Razurel et al., 2011; Allott et al., 2015; Suzuki et al., 2018); the difference in coping strategies used by athletes is related to their competence to the understanding of sports, in which competence perception is an important determinant in secondary assessments, and social support can have an indirect effect on avoidance responses through reduced threat assessments and increased control assessments (secondary assessment) (Hudek-Knezevic and Kardum, 1996; Pajares and Cheong, 2003; Slattery et al., 2013). Besides, a large amount of research has supported that the coping strategies adopted by individuals faced with stress have a strong relationship with social support and that adaptive and proactive coping strategies are also highly related to the increase in social support (Shulman, 1993; Heijmans, 1998; Nicolas, 2009).

In short, most of the scholars in the previous research (Bandura, 1977; Antonio and Alexandre, 2017; Pulido et al., 2018; Ponseti et al., 2019; de Francisco et al., 2020; Prats et al., 2020) considered the primary assessment, secondary assessment, and implementation of coping strategies as the same processing stage, so it is impossible to see whether the primary assessment and the secondary assessment play an intermediary role in the stress coping model theory, and in the stress coping theoretical model, the generation of primary and secondary assessments follow a chronological order. Secondly, the previous scholars’ coping strategies for problem focus and emotional focus are both proactive and positive coping responses. In the field of competitive sports, the problem focus and emotional focus are combined into a positive response, and the evasion strategy is called negative coping. The suitability of this treatment is also worthy of further investigation. Based on this, this research takes middle school students’ campus soccer players as the survey samples and discusses the applicability of this model in the sports field based on the relevant theories of the stress coping execution model. Therefore, the following research hypotheses were proposed: (1) Primary assessment in social support and competence perception plays a mediating role in the influence of secondary assessment; (2) Secondary assessment plays an intermediary role in the influence of social support and competence perception on stress coping strategy; (3) The mode and actuality of the strategy for coping with stress in the competition proposed by this research has an acceptable fit with the observed data.

## Subjects and Methods

### Subjects

“PXT” 3 levels Campus League is a new campus soccer league scheme launched by the Sichuan Campus Soccer Association. P represents the popular campus soccer league; X represents the campus soccer tournament; T represents the advanced campus soccer league. Teams from U9 to U17 will participate in the competition according to each age group, with 5, 8, and 11 players, respectively. There are currently 600 teams participating in the primary school league and 50 teams in the secondary school league.

In this study, 396 male athletes from 22 teams participating in the 5th Chengdu High School Men’s Soccer Game in Chengdu were surveyed. Perception, cognitive assessment, and pre-competition stress coping strategies were investigated to reveal the relationship between these variables. A total of 396 questionnaires were sent out, 41 invalid copies were excluded, and 355 valid copies remained. After the definition of the stress experience confirmation scale, 34 athletes with scores of five or less, indicating to have insufficient or no awareness of pressure in the competition, were eliminated. Finally, the copies obtained in this study were 331. Among them, 172 students are in the 10th school grade, and 159 students are in the 11th school grade. The average age of subjects was 16.52 ± 0.78 years old, height was 174.45 ± 6.87 cm, and body mass was 65.87 ± 5.81 kg.

All participants provided written informed consent, and this study was in accordance with the local laws and regulations.

### Measurement Tools

#### The Perceived Stress Reactivity Scale for Adolescent Athletes

The stress experience confirmation scale developed by Britton et al. (2017) was used. The purpose of this scale was to test whether the players can experience stress in the competition. Based on the experience of the athletes in the last 6 months, the scale selected “Yes” or “No” for the stress situation described in each item. There were 14 items on the full scale. If they selected “Yes,” they got one point; if they selected “No,” they got 0 points. If the score was less than five points, it meant that the participants were not aware of pressure or lack of pressure, and the participant should be excluded.

#### The Social Support Scale

This study has revised the sports social support scale compiled by Ku et al. (2010). The total scale had 13 items in two dimensions, namely, emotional support (seven items) and information support (six items). The items used a four-point Likert-type scale (“never” for one point, “always” for four points). Every item in both dimensions included three sources of support, namely, parents, peers, and teachers.

#### The Ability to Perception Scale

This study used the ability perception scale compiled by Martinent and Ferrand (2007). There were three items in this study. They measured the individual’s ability to evaluate specific exercise compared with others. Through the six-point scale, only both ends were marked with semantic meaning, 1 = very poorly, 6 = very well, respectively.

#### The Cognitive Assessment Scale

This study used the cognitive assessment scale developed by Lai (2006), which included two subscales, namely primary assessment and secondary assessment. There were 21 items in the primary assessment full scale, of which the positive assessment was the challenge assessment (four items), and the negative assessment included the injury assessment (10 items) and the threat assessment (seven items). The five-point scale method was adopted, and only the semantic meaning was marked at the two ends, which were 1 = no such feeling, 5 = powerful feeling. There were four descriptions of the secondary assessment scale, which were (1) I have no way to change this situation, so give up any possible attempts. (2) For the current situation, I think I have to accept. (3) I need some instructions to deal with the current situation. (4) In general, I feel I have the ability to change or control the situation. Scoring criteria: 1 for one point, 2 for two points, and so on.

#### The Competition Stress Coping Strategy Scale

The competition pressure coping strategy scale developed by Lai (2006) was used. There was a 22-item scale. The positive coping included four dimensions: proactive (6 items), concentration (6 items), seeking support (four items), and bright side (four items). Negative coping included avoidance (two items). The positive coping intended to positively deal with the stress environments, to hope to remove or mitigate the impact of the stressful situation; negative avoidance intended to avoid the problem, no longer try to deal with the stressful situation, ignore it or even deny the creation of stressful situations. The scale used a 5-point scoring method. If it was not used at all, it was one point, and always used, five points.

### The Validity and Reliability Test of Scales

Table 1 shows:

**TABLE 1:** Quality analysis of four measurement scales.

**Scales**	**Dimension naming**	**KMO and Bartlett**	**Items**	**Explained variance %**	**Accumulated explained variance %**	**Combined confidence CR**	**Cronbach’s α**
Social support	Emotional support	KMO = 0.84; *P* < 0.05	7	35.15	35.15	0.84	0.84
	Information support		6	21.44	56.59	0.80	0.85
Measurement model verification outcomes: AGFI = 0.95, CFI = 0.98, NFI = 0.91, IFI = 0.97, RMSEA = 0.034; Overall Cronbach’s α = 0.87							
Ability perception		KMO = 0.83; *P* < 0.05	3	67.64	67.64	–	0.85
Measurement model verification outcomes: AGFI = 0.94, CFI = 0.99, NFI = 0.96, IFI = 0.96, RMSEA = 0.029; Overall Cronbach’s α = 0.85							
Primary assessment	Positive assessment	KMO = 0.92; *P* < 0.05	4	41.17	41.17	0.83	0.81
	Negative assessment		17	33.24	74.41	0.87	0.89
Measurement model verification outcomes: AGFI = 0.97, CFI = 0.98, NFI = 0.95, IFI = 0.95, RMSEA = 0.025; Overall Cronbach’s α = 0.85							
Stress coping strategy for competition	Positive coping	KMO = 0.84; *P* < 0.05	20	47.25	47.25	0.83	0.86
	Negative coping		2	17.66	64.91	0.89	0.91
Measurement model verification outcomes: AGFI = 0.93, CFI = 0.98, NFI = 0.96, IFI = 0.99, RMSEA = 0.030; Overall Cronbach’s α = .87							

(1) The 13-item social support scale was very suitable for factor analysis (KMO = 0.84; *P* < 0.05). The explanatory power of the two common factors (emotion support and information support) was 35.15 and 21.44%, respectively, with an accumulated contribution rate of 56.59%. In terms of reliability, the Cronbach’s α coefficients of the two factors were 0.84 and 0.85, respectively, and the overall Cronbach’s α coefficient of the scale was 0.87; the measurement model fit index of AGFI, CFI, NFI, and IFI were 0.95, 0.98, 0.91, and 0.97, respectively. All of which was greater than the good fit criteria of 0.90, RMSEA = 0.034 (<0.05 for a good fit); In addition, the latent variables of the combined reliability CR of the two common factors was 0.84 and 0.80, respectively, which showed that the scale had excellent reliability and validity.

(2) The Cronbach’s α coefficient of the three-item ability perception scale was 0.85. In the test of the fit of the measurement model, AGFI = 0.94, CFI = 0.99, NFI = 0.96, and IFI = 0.96, all of which were greater than the good fit criteria of 0.90, and RMSEA = 0.029 (less than 0.05 is a good fit). It showed that the scale had good reliability and validity.

(3) The 21-item primary assessment scale can be extracted from two common factors (positive assessment and negative assessment) by exploratory factor analysis (EFA) (KMO = 0.83; *P* < 0.05), and their explanatory power was 41.17 and 33.24%, respectively, with an accumulated contribution rate of 74.41%. In terms of reliability, the Cronbach’s α coefficients of the two common factors were 0.81 and 0.89, respectively, and the overall Cronbach’s α coefficient was 0.85; the measurement model fit index of AGFI, CFI, NFI, and IFI were 0.97, 0.98, 0.95, and 0.95, respectively, all of which was greater than the goodness of fit criteria of 0.90, RMSEA = 0.025 (less than 0.05 for a good fit). In addition, the combined reliability CR of the latent variables of two common factors was 0.83 and 0.87, which showed that the scale had excellent reliability and validity.

(4) The 22-item competitive stress coping scale can be extracted two common factors (positive coping and negative coping) by EFA (KMO = 0.84; *P* < 0.05). The explanatory power was 47.25 and 17.66%, respectively, with an accumulated contribution rate of 64.91%. In terms of reliability, the Cronbach’s α coefficients of the two common factors were 0.86 and 0.91, respectively, and the overall Cronbach’s α coefficient was 0.87; the measurement model fit index of AGFI, CFI, NFI, and IFI were 0.93, 0.98, 0.96, and 0.99, respectively, all of which was greater than the goodness of fit criteria of 0.90, RMSEA = 0.030 (less than 0.05 for a good fit). In addition, the combined reliability CR of the latent variables of two common factors was 0.83 and 0.89, which showed that the scale had excellent reliability and validity.

### Statistical Analysis

After the questionnaires were collected, they were encoded, and their database has been built. Statistical analysis was performed using SPSS Statistics 21.0 and AMOS 21.0 software packages. In addition to descriptive statistics and correlation analysis, this study used EFA and confirmatory factor analysis (CFA) to reveal the causal relationship between related variables. The significant level of all indicators was set to α = 0.05.

## Results

### Descriptive Statistics and Correlation Analysis

Table 2 shows:

**TABLE 2:** Matrix table of correlation coefficient of various variables.

**Variables**	**SS**	**AP**	**NA**	**PA**	**SA**	**PC**	**NC**
SS	1.00						
AP	0.25**	1.00					
NA	−0.02	−0.23**	1.00				
PA	0.35**	0.08	0.29**	1.00			
SA	0.23**	0.26**	−0.27**	0.25**	1.00		
PC	0.51**	0.27**	0.03	0.44**	0.32**	1.00	
NC	0.06	0.01	0.07	−0.18*	−0.05	0.01	1.00
SD	0.71	0.87	0.84	0.91	0.88	0.94	0.81
Mean	2.82	3.49	2.88	3.56	3.28	3.26	3.28

SS, social support; AP, ability perception; NA, negative assessment; PA, positive assessment; SA, secondary assessment; PC, positive coping; NC, negative coping; SD, standard deviation. *p < 0.05 and **p < 0.01.

Positive significant correlations were observed between social support and ability perception (*r* = 0.25, *p* < 0.01), positive evaluation (*r* = 0.35, *p* < 0.01), secondary assessment (*r* = 0.23, *p* < 0.01), and positive coping (*r* = 0.51, *p* < 0.01); ability perception was significantly negatively correlated with negative assessment (*r* = −0.23, *p* < 0.01), and significantly positive correlated with secondary assessment (*r* = 0.26, *p* < 0.01) and positive coping (*r* = 0.27, *p* < 0.01), suggesting that when players perceived more social support (emotion and information support) and higher ability perceptions, the more stress has been perceived as a challenge, and the less stress has been perceived as a negative assessment (injury or threat). In the coping strategy, the negative assessment was significantly positively correlated with the positive assessment (*r* = 0.29, *p* < 0.01), but was significantly negatively correlated with the secondary assessment (*r* = −0.27, *p* < 0.01); the positive assessment was significantly correlated with the secondary assessment (*r* = 0.25, *p* < 0.01) and the positive coping (*r* = 0.44, *p* < 0.01) but was significantly negatively correlated with the negative coping (*r* = −0.18, *p* < 0.01). Positive coping was significantly positively correlated (*r* = 0.32, *p* < 0.01). This meant that the more the players regard stress as a challenge, and the more likely they were to control or change stress events, the more they tended to use positive coping strategies.

### The Mediating Effect of Primary Assessment on the Influence of Social Support and Ability Perception on Secondary Assessment

According to Kendall and Terry’s (2009) regression method to estimate the mediating effect, three prediction models were constructed in this study. Model 1 was named the criterion variable model, model 2 was the mediating variable model, and model 3 was the criterion + mediating variable model.

### Speculation Model Test of the Mediating Effect of Primary Assessment

Table 3 shows:

**TABLE 3:** The fit measures of model assessment.

	**Absolute fit test**						**Value-added fit test**			
**Models**	**X^2^**	**X^2^/df**	**P**	**RMSEA**	**GFI**	**AGFI**	**NFI**	**IFI**	**CFI**	**RFI**
Model 1	0.00	0.00	0.99	0.00	1.00	1.00	1.00	1.00	1.00	1.00
Model 2	1.74	1.74	0.19	0.05	1.00	0.97	0.98	0.99	0.99	0.91
Model 3	0.52	0.26	0.77	0.00	1.00	1.00	1.00	1.00	1.00	0.99

*P < 0.05 indicates that the model is not acceptable fit; P > 0.05 indicates that it is acceptable fit.*

(1) From the results of the absolute fit test: the absolute fit indexes of models 1, 2, and 3 of X^2^/df were 0.00, 1.74, and 0.26, respectively, and the corresponding probabilities P were 0.99, 1.74, and 0.26, respectively, all of which were <0.05, indicating three covariance matrices of the hypotheses model were well fit to the observed data (generally, the value of X^2^/df should be between 1 and 3 for a good fit, and between 0–1 is the best fit); the GFI values of the three models were 1.00, 1.00, and 1.00 (>0.90 for a good fit), AGFI values were 1.00, 0.97, and 1.00 (>0.90 for good fit), RMSEA was 0.00, 0.05, and 0.00 (RMSEA < 0.05 is excellent, 0.05–0.08 is good).

(2) From the results of the value-added fit test: the NFI values of the three models were 1.00, 0.98, and 1.00 (>0.90 for acceptable fit), respectively; the IFI were 1.00, 0.99, and 1.00 (>0.90 for acceptable fit), respectively; the CFI values were 1.00, 0.99, and 1.00 (>0.90 for acceptable fit), respectively; the RFI values were 1.00, 0.91, and 0.99 (>0.90 for acceptable fit), respectively. In short, whether it was an absolute fit or an increment fit test, the hypotheses models 1, 2, and 3 in this study had better fit to observed data.

### Estimation of Mediating Effects of Primary Assessment

**FIGURE 1:**
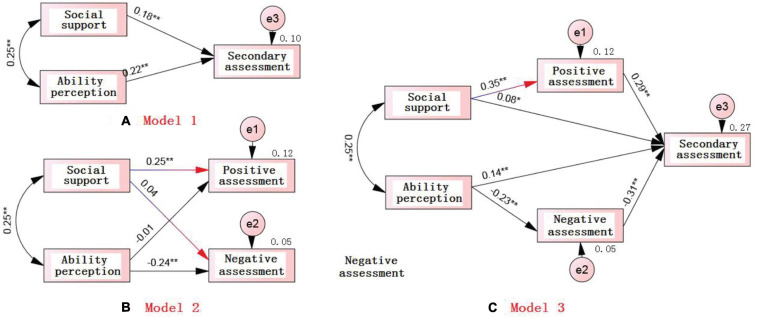
The effects of social support and ability perception on primary and secondary assessments: **(A)** Model 1: Direct effects of social support and ability perception on primary assessment; **(B)** Model 2: Direct effects of social support and ability perception on secondary assessments; **(C)** Model 3: Effects of social support and ability perception on secondary assessments *via* the mediating role of primary assessments.

In this study, the coping strategies of the problem and emotional focus were incorporated into the positive coping strategies, that is, they were merged into positive coping strategies, and the avoidance strategies were named negative coping strategies. The three path model diagrams (model 1, model 2, and model 3) indicate:

Step 1: The structural equation model 1 (Figure 1A) showed that social support and ability perception had a direct effect on the secondary assessment, and the normalization coefficients R of their influence paths were 0.18 and 0.22, respectively;

Step 2: The regression model 2 (Figure 1B) indicated that the social support had a significant direct influence on positive assessment and the ability perception also had a significant direct influence on negative assessment (the primary assessment was made up of positive and negative assessments), and the influence path normalization coefficients of R were 0.25 and −0.24, respectively.

Step 3: The regression model 3 (Figure 1C) indicated that the normalization coefficient of the direct influence of the path of social support on the secondary assessment had become 0.08 (insignificant). In contrast, the direct impact in model 1 was 0.18, which indicated the positive assessment acted as a full mediating role between social support and the secondary assessment, with an influence of 0.35 × 0.29 = 0.11 (that is, the 11% variation in the second assessment can be explained); on the other hand, the normalized path coefficient of the direct impact of the ability perception in model 3 on the secondary assessment was 0.14 (still significant), but it had a significant downward trend compared with 0.22 of the normalization coefficient of the direct impact path of the model in model 1, so it can be inferred that the secondary assessment acted as partial mediation, with a mediating effect of 0.23 × 0.31 = 0.08 (that is, 8% of the variation in the secondary assessment).

### Mediating Effects of Secondary Assessments on Perceived Social Support, Ability Perception, and Predictive Coping Strategies of Primary Assessment

#### Validation of the Secondary Assessment Intermediary Model

Table 4 shows:

**TABLE 4:** Statistical table of the degree of fit for model assessment.

	**Absolute fit test**						**Value-added fit test**			
**Models**	**X^2^**	**X^2^/df**	**P**	**RMSEA**	**GFI**	**AGFI**	**NFI**	**IFI**	**CFI**	**RFI**
Model 4	2.72	1.36	0.26	0.034	1.00	0.97	0.99	1.00	0.99	0.93
Model 5	10.64	1.52	0.16	0.040	0.99	0.96	0.97	0.99	0.99	0.91

*P < 0.05 indicates that the model is not fit; P > 0.05 indicates that it is fit.*

(1) The results from the absolute fit test: the absolute fit indexes X^2^/df of models 4 and 5 were 1.36 and 1.52, respectively. The corresponding probabilities P were 0.26 and 0.16, which were <0.05, indicating that the covariance matrices of models 4 and 5 were well fit to the observed data (generally, the X^2^/df value should be between 1 and 3, and between 0 and 1 was very fit); the GFI values of the two models were 1.00 and 0.99 (>0.90 is acceptable fit), the AGFI values were 0.97 and 0.96, respectively (>0.90 was acceptable fit), the RMSEA was 0.034 and 0.040, respectively, (generally, RMSEA < 0.05 is excellent, and 0.05–0.08 is good).

(2) In the value-added fit test results, the two models had NFI values of 0.99 and 0.96 (>0.90 for acceptable fit), IFI values were 1.00 and 0.99, respectively (>0.90 for acceptable fit), and CFI values were 0.99 and 0.99 (>0.90 for acceptable fit), the RFI values were 0.93 and 0.91, respectively (>0.90 for acceptable fit). In short, whether it was an absolute or increment fit test, it was assumed that the model 4 and 5 covariance matrices fitted well with the observed data.

#### Estimation of Mediating Effects of Secondary Assessment

**FIGURE 2:**
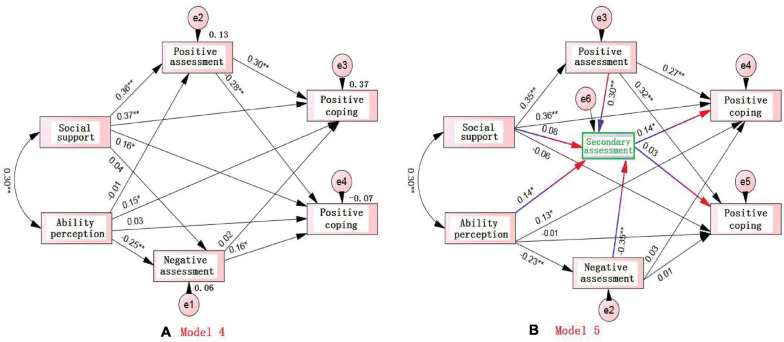
Effects of social support, ability perception, and primary assessment on competition coping strategies: **(A)**: Model 4: Direct effects of social support, ability perception, and primary assessment on competition coping strategies; **(B)** Model 5 Effects of social support, ability perception, and primary assessment on competition coping strategies *via* mediating role of secondary assessment.

From the path diagrams of models 3, 4, and 5, (Figures 1C, 2A,B) we can see:

Step 1: According to the path diagrams of the secondary assessment mediation model 4, it can be seen that the positive primary assessment (referred to as positive assessment) can significantly predict the positive coping (standardized path coefficient was 0.30^∗∗^), and it can also significantly predict the negative coping (standardized path coefficient was −0.28^∗∗^), while the negative primary assessment (referred to as negative assessment) can significantly predict negative coping (standardized path coefficient was 0.16) but cannot predict positive coping; social support can significantly predict positive coping (normalized path factor 0.37), can also significantly predict negative coping (normalized path factor was 0.16), ability perception can significantly predict positive coping (normalized path factor was 0.15), but cannot predict negative coping (normalized path factor was 0.03).

Step 2: The path diagram of the secondary assessment mediation model 3 has clearly shown that the positive assessment can significantly predict the secondary assessment (standardized path factor was 0.29^∗∗^), while the negative assessment can also significantly predict the secondary assessment (standardized path factor was −0.31).

Step 3: From the path diagram of the secondary assessment intermediary model 5 that the positive primary assessment had a direct downward effect on the positive coping (from 0.30 to 0.27), which meant that the secondary assessment played the partial mediating role between positive assessment and positive coping, with a mediating influence of 0.31 × 0.14 = 0.05 (that is, explaining 5% variation of positive coping); positive assessment predicted that the secondary assessment would reach a significant level (the standardized path coefficient was 0.30), and the direct effect on the negative coping has reached a significant level (The standardized path coefficient was 0.32, *p* < 0.01), but the direct effect of the secondary assessment on the negative coping has not reached a significant level (the standardized path coefficient was 0.03, not significant), so the secondary assessment was not an intermediary variable between positive assessment and negative coping; negative assessment in model 4 could significantly predict negative coping, but the negative assessment on negative coping in model 5 has almost disappeared (the standardized path coefficient was 0.00), but the negative assessment had a significant impact on the secondary assessment (the standardized path coefficient was −0.35), so it can be inferred that the secondary assessment played the full mediating role between the negative assessment and the positive coping, with a mediating effect of 0.35 × 0.14 = 0.05 (explaining 5% of the variation of positive coping). Because social support could not predict secondary assessments, secondary assessments would not act as an intermediary between social support and positive coping, while the predicting effect of ability perception on positive coping in model 3 (0.14^∗^) has not changed significantly in model 5 (beta = 0.13, *p* < 0.05), despite the significant predicting power of ability perception on secondary assessments (the standardized path factor was 0.14), so it can be inferred that the secondary assessments were also not an intermediary between ability perception and positive coping.

## Analysis and Discussion

### The Mediating Effect of Primary Assessment on the Influence of Social Support and Ability Perception on Secondary Assessment

Personal factors (such as personal beliefs, motivation goals, commitments, and values) and situational factors (such as environmental needs, resources, social constraints, and secular perspectives) affect an individual’s cognitive assessment, and the individual’s cognitive assessment determines the individual’s coping strategies, assessments and coping affect individual adaptation (Nicholls et al., 2016; Delahaij and van Dam, 2017; Helbig and Backhaus, 2017). Cognitive assessment is an evaluation process, which includes three stages: primary assessment, secondary assessment, and re-assessment. Many scholars have previously divided primary assessment and secondary assessment into the same stage without distinction. In fact, in the stress coping model, the generation of primary assessment and secondary assessment is chronological. Therefore, this study is more appropriate to discuss the primary assessment and secondary assessment according to the stage of generation.

This study considered perceived social support as situational factors and ability perception as individual factors. It explored the prediction of social support, ability perception, and primary assessment to the secondary assessment, so as to reveal whether primary assessment played a partial mediating effect between social support, ability perception, and secondary assessment. It was found that positive primary assessment (referred to as positive assessment) has a full mediating effect on social support and secondary assessment, that is, social support will affect secondary assessment through positive assessment. In other words, the more social support a player receives, the more positive his assessment will be in the face of a stressful event, which will, in turn, affect the player’s assessment that he is better able to change or control the stressful event. This is consistent with the results of previous studies by many scholars (Muller et al., 2000; Ommen et al., 2008), that is, individuals with more social support perceive that negative events are relatively less threatening to individuals, and therefore they are more likely to face stress with a positive attitude and tend to make positive assessments. Besides, this study found that negative primary assessment (referred to as negative assessment) acted as a partial mediator between ability perception and secondary assessment, that is, ability perception can affect secondary assessment through negative assessment. It is manifested that the higher the player’s ability perception is, the less the negative assessment will be generated when facing a stress event, which will affect the player’s assessment that he can change or control the stress event. Third, this study did not find that social support can influence secondary assessments through negative assessments, nor that ability perception can affect sub-assessments through positive assessments, which supports the views of some previous scholars (Kaiseler et al., 2009; Jordan et al., 2015; Nicholls et al., 2016).

### Mediating Effects of Secondary Assessments on Social Support, Ability Perception, and Primary Assessment Prediction Coping Strategies

The results of this study showed that secondary assessment was not an intermediary of perceived social support, ability perception, and positive coping strategies. Similarly, the secondary assessment was not an intermediary of perceived social support, ability perception, and negative coping strategies. However, the secondary assessment served as a part of the intermediary effect between the positive assessment and the positive coping strategy. In other words, the positive assessment can influence the positive coping strategy through the secondary assessment, that is, when the assessment of the athlete facing a stress event was more positive, it can prompt them to evaluate that they are better able to change or control stressful events, and then adopt a positive and active causal strategy. This finding was consistent with the results of some previous scholars (Terry, 1991; Das et al., 2017) but different from the results of other scholars (Firk and Markus, 2009; Dunkley et al., 2017). Dunkley et al. (2017) have found from the path analysis that the secondary assessment of stress events was not an intermediary between positive assessment and positive coping; that is, the primary assessment did not affect the coping strategy through the secondary assessment but direct coping strategy. Therefore, this study believed that future research needs to develop a secondary assessment scale to explore the relationship between the primary assessment and the secondary assessment to improve the entire stress coping model.

### The Causal Model of the Overall Coping Strategy of Competitive Stress

From the perspective of the overall model structure, perceived social support has significant predictive power on both positive assessment (beta = 0.35, *p* < 0.01) and positive coping strategies (beta = 0.36, *p* < 0.01), and ability perception had significant predictive power on secondary assessment, negative assessment, and positive coping (beta = 0.14, *p* < 0.05; beta = −0.23, *p* < 0.01, beta = 0.13, *p* < 0.05). These results implied that perceived social support and ability perception played important roles in the positive assessment and coping strategies of athletes in coping with competitive stress; however, this study found that perceived social support had little effect on secondary assessments and negative coping strategies. Ability perception had a significant predictive effect on secondary assessments and negative coping strategies, while some previous scholars (Shulman, 1993; Ramdhani et al., 2015; Shnaider et al., 2017) have argued that perceived social support has a negative impact on secondary assessments and negative coping. There is a significant predictive power to coping strategies, and these inconsistencies need to be further explored in the future. Besides, this study found that the positive assessment has significant predictive power for both the secondary assessment and the positive and negative coping strategies (beta = 0.30, *p* < 0.01; beta = 0.27, *p* < 0.05, beta = 0.32, *p* < 0.01), and the negative assessment had significant predictive power on secondary assessment (beta = −0.35, *p* < 0.01), and secondary assessments had significant predictive power on positive coping strategies (beta = 0.14, *p* < 0.05). The above results implied that the positive and negative primary and secondary assessment played important roles in the player’s competitive stress coping strategy. The positive primary assessment had the strongest influence on the negative coping strategy, followed by a positive coping strategy. These results were consistent with the results of some previous studies (Lai, 2006; Britton et al., 2017), which implied that the player’s challenge of assessment and the more active secondary assessment could help individuals not only to avoid the problem, and use positive coping to deal with the problem. It also implies that a positive primary assessment of positive coping strategy is more positive than a negative primary assessment and secondary assessment.

## Conclusion, Limitations, and Recommendations

### Conclusion

(1) The competitive stress coping strategy model has a good degree of fit to the actual observed data, of which the primary assessment and the secondary assessment play different roles in the stress coping mode theory;
(2) The more perceived social support athletes being received, the more positive its assessment in the face of stressful events is, which is conducive to players’ assessment of their ability to change or control stressful events;
(3) The higher the ability to perception in athletes, the less likely they are to experience negative stresses. To the assessment, which in turn affects the players’ assessment of their ability to change or control stress events;
(4) When athletes face stress events, the more they tend to regard stress as a positive assessment, the more they will adopt a positive attitude to deal with stress, and use positive coping strategies.

### Limitations

(1) Because the present study was a cross-sectional study, it cannot thoroughly explore the impact of perceived social support and ability perception on coping strategies for competitive stress in soccer players. The longitudinal study should be carried out in a future study.
(2) Lazarus and Folkman’s transactional model of stress and coping should be added into the future study.
(3) The secondary assessment scale in this study only used one item to explore. This may affect the relationship between the primary assessment and the secondary assessment, as well as the adaptability of the overall competition stress coping model. Therefore, it is necessary for future researchers to develop the secondary assessment scale further and explore the relationship between the primary assessment and the secondary assessment to complete the entire stress-coping model.

### Recommendations

(1) For competitive stress coping strategy, covariate variables such as athletic performance, sports satisfaction, etc., can be added into competitive stress coping in the future study, in doing so, to have a deeper understanding of the competitive stress coping strategy.
(2) This study explored the relationship between the cognitive assessment of athletes during the competition with the pressure of soccer players. In the future, ordinary practice or stress events in daily life can be added. Because for athletes the automated cognitive assessment formed by ordinary practice may have a greater impact on athletes’ coping strategies and adaptation, future research may consider exploring the relationship between stress events and cognitive assessment of ordinary practice.

## Data Availability Statement

The original contributions presented in the study are included in the article/supplementary material, further inquiries can be directed to the corresponding author/s.

## Ethics Statement

Ethical review and approval was not required for the study on human participants in accordance with the local legislation and institutional requirements. Written informed consent to participate in this study was provided by the participants’ legal guardian/next of kin.

## Author Contributions

ZD carried out the survey. ZD and QL analyzed the data. ZD and WM wrote the first draft. CY revised this manuscript. All authors designed the study and reviewed the manuscript.

## Conflict of Interest

The authors declare that the research was conducted in the absence of any commercial or financial relationships that could be construed as a potential conflict of interest.

## References

[B1] Alhurani A. S. Dekker R. Ahmad M. Miller J. Debra K. Moser D. K. (2018). Stress, cognitive appraisal, coping, and event free survival in patients with heart failure. *Heart Lung* 47 205–210. 10.1016/j.hrtlng.2018.03.008 29627073 PMC5924439

[B2] Allott K. A. Rapado-Castro M. Proffitt T. M. Bendall S. Phillips L. J. (2015). The impact of neuropsychological functioning and coping style on perceived stress in individuals with first-episode psychosis and healthy controls. *Psychiatry Res.* 226 128–135. 10.1016/j.psychres.2014.12.032 25618467

[B3] Antonio N. P. Alexandre G. M. (2017). Relationship between performance and anxiety in sports: a systematic review. *Retos* 32 172–177. 10.47197/retos.v0i32.53297

[B4] Azizi M. (2011). Effects of doing physical exercises on stress-coping strategies and the intensity of the stress experienced by university students in Zabol, Southeastern Iran. *Procedia Soc. Behav. Sci.* 30 372–375. 10.1016/j.sbspro.2011.10.073

[B5] Bandura A. (1977). Self-efficacy: toward a unifying theory of behavioral change. *Psychol. Rev.* 84 191–215. 10.1016/0146-6402(78)90002-4 847061

[B6] Britton D. Kavanagh E. Polman R. (2017). The perceived stress reactivity scale for adolescent athletes. *Pers. Individ. Differ.* 116 301–308. 10.1016/j.paid.2017.05.008

[B7] Das S. Das B. Nath K. Dutta A. Hazarika M. (2017). Impact of stress, coping, social support, and resilience of families having children with autism: a North East India-based study. *Asian J. Psychiatry* 28 133–139. 10.1016/j.ajp.2017.03.040 28784366

[B8] de Francisco C. Parra-Plaza F. J. Vílchez P. M. (2020). Psychological needs in Spanish athletes: validation of the “Basic Needs Satisfaction in Sport Scale”. *Apunts. Educ. Física Deportes* 141 11–20. 10.5672/apunts.2014-0983.es.(2020/3).141.02

[B9] Delahaij R. van Dam K. (2017). Coping with acute stress in the military: the influence of coping style, coping self-efficacy and appraisal emotions. *Pers. Individ. Differ.* 119 13–18. 10.1016/j.paid.2017.06.021

[B10] Dunkley D. M. Lewkowski M. Lee I. A. Preacher K. J. Westreich R. (2017). Daily stress, coping, and negative and positive affect in depression: complex trigger and maintenance patterns. *Behav. Ther.* 48 349–365. 10.1016/j.beth.2016.06.001 28390498

[B11] Firk C. Markus C. R. (2009). Mood and cortisol responses following tryptophan-rich hydrolyzed protein and acute stress in healthy subjects with high and low cognitive reactivity to depression. *Clin. Nutr.* 28 266–271. 10.1016/j.clnu.2009.03.002 19345451

[B12] Gao W. Ping S. Liu X. Q. (2020). Gender differences in depression, anxiety, and stress among college students: a longitudinal study from China. *J. Affect. Disord.* 263 292–300. 10.1016/j.jad.2019.11.121 31818792

[B13] Gourounti K. Anagnostopoulos F. Potamianos G. Lykeridou K. Vaslamatzis G. (2012). Perception of control, coping and psychological stress of infertile women undergoing IVF. *Reprod. Biomed. Online* 24 670–679. 10.1016/j.rbmo.2012.03.002 22503340

[B14] Goyen M. J. Anshel M. H. (1998). Sources of acute competitive stress and use of coping strategies as a function of age and gender. *J. Appl. Dev. Psychol.* 19 469–486. 10.1016/S0193-3973(99)80051-3

[B15] Hayward F. P. I. Knight C. J. Mellalieu S. D. (2017). A longitudinal examination of stressors, appraisals, and coping in youth swimming. *Psychol. Sport Exerc.* 29 56–68. 10.1016/j.psychsport.2016.12.002

[B16] Heijmans M. J. W. M. (1998). Coping and adaptive outcome in chronic fatigue syndrome: importance of illness cognitions. *J. Psychosom. Res.* 45 39–51. 10.1016/S0022-3999(97)00265-1 9720854

[B17] Helbig S. Backhaus J. (2017). Sex differences in a real academic stressor, cognitive appraisal and the cortisol response. *Physiol. Behav.* 179 67–74. 10.1016/j.physbeh.2017.05.027 28546084

[B18] Hudek-Knezevic J. Kardum I. (1996). A model of coping with conflicts between occupational and family roles: structural analysis. *Pers. Individ. Differ.* 21 355–372. 10.1016/0191-8869(96)00055-4

[B19] Jordan E. J. Vogt C. A. DeShon R. P. (2015). A stress and coping framework for understanding resident responses to tourism development. *Tour. Manag.* 48 500–512. 10.1016/j.tourman.2015.01.002

[B20] Kaiseler M. Polman R. Nicholls A. (2009). Mental toughness, stress, stress appraisal, coping and coping effectiveness in sport. *Pers. Individ. Differ.* 47 728–733. 10.1016/j.paid.2009.06.012

[B21] Kendall E. Terry D. (2009). Predicting emotional well-being following traumatic brain injury: a test of mediated and moderated models. *Soc. Sci. Med.* 69 947–954. 10.1016/j.socscimed.2009.06.021 19616354

[B22] Ku C. M. Lin H. E. Chen W. C. (2010). Scale establishment for the exercise social support of college students. *J. I. Tour. Res.* 2 80–101. 10.29859/JITR.201001.0005

[B23] Lai W. X. (2006). *The Relationships of Social Support and Perceived Competence to Cognitive Appraisals and Coping Strategies in High School Athletes*. Unpublished Master’s thesis. Taipei: Department of physical education, National Taipei University of Education. 10.6344/NTUE.2006.00030

[B24] Lazarus R. S. Folkman S. (1984). *Stress, Appraisal, and Coping.* New York, NY: Springer.

[B25] Ma J. Roca-Chiapas D. I. Solís-Ortiz S. Fajardo-Araujo M. Sosa M. Rosa-Zarate A. (2010). Stress profile, coping style, anxiety, depression, and gastric emptying as predictors of functional dyspepsia: a case-control study. *J. Psychosom. Res.* 68 73–81. 10.1016/j.jpsychores.2009.05.013 20004303

[B26] Maltby J. Day L. (2003). Religious orientation, religious coping and appraisals of stress: assessing primary appraisal factors in the relationship between religiosity and psychological well-being. *Pers. Individ. Differ.* 34 1209–1224. 10.1016/S0191-8869(02)00110-1

[B27] Martinent G. Ferrand C. (2007). A cluster analysis of precompetitive anxiety: relationship with perfectionism and trait anxiety. *Pers. Individ. Differ.* 43 1676–1686. 10.1016/j.paid.2007.05.005

[B28] Muller R. T. Goebel-Fabbri A. E. Diamond T. Dinklage D. (2000). Social support and the relationship between family and community violence exposure and psychopathology among high risk adolescents. *Child Abuse Negl.* 24 449–464. 10.1016/S0145-2134(00)00117-4 10798836

[B29] Nicholls A. R. Levy A. R. Carson F. Thompson M. A. Perry J. L. (2016). The applicability of self-regulation theories in sport: goal adjustment capacities, stress appraisals, coping, and well-being among athletes. *Psychol. Sport Exerc.* 27 47–55. 10.1016/j.psychsport.2016.07.011

[B30] Nicolas M. (2009). Personality, social support and affective states during simulated microgravity in healthy women. *Adv. Space Res.* 44 1470–1478. 10.1016/j.asr.2009.07.013

[B31] Olmedilla A. Ortega E. Robles-Palazón F. J. Salom M. García-Mas A. (2018). Healthy practice of female soccer and futsal: identifying sources of stress, anxiety and depression. *Sustainability* 10:2268. 10.3390/su10072268

[B32] Ommen O. Janssen C. Neugebauer E. Bouillon B. Pfaff H. (2008). Trust, social support and patient type: associations between patients perceived trust, supportive communication and patients preferences in regard to paternalism, clarification and participation of severely injured patients. *Patient Educ. Couns.* 73 196–204. 10.1016/j.pec.2008.03.016 18450408

[B33] Pajares F. Cheong Y. F. (2003). Achievement goal orientations in writing:a developmental perspective. *Int. J. Educ. Res.* 39 437–455. 10.1016/j.ijer.2004.06.008

[B34] Ponseti F. J. Almeida P. L. Lameiras J. Martins B. Olmedilla A. López-Walle J. (2019). Self-determined motivation and competitive anxiety in athletes/students: a probabilistic study using Bayesian networks. *Front. Psychol.* 10:1947. 10.3389/fpsyg.2019.01947 31555166 PMC6742710

[B35] Prats A. N. Verdaguer F. Abad A. S. Mas A. G. (2020). Anxiety and perceived performance in athletes and musicians: revisiting Martens. *Rev. Psicol. Deporte* 29 21–28.

[B36] Pulido D. Borràs P. A. Salom M. Ponseti F. J. (2018). Competitive anxiety in grassroots sport in the Balearic Islands. *Rev. Psicol. Deporte* 27 13–17.

[B37] Ramdhani N. Widjaja J. D. Rahmawati N. (2015). Internet supported cognitive behavior therapy to help students with shy-socially isolated problems. *Procedia Soc. Behav. Sci.* 165 179–188. 10.1016/j.sbspro.2014.12.620

[B38] Razurel C. Bruchon-Schweitzer M. Dupanloup A. Irion O. Epiney M. (2011). Stressful events, social support and coping strategies of primiparous women during the postpartum period: a qualitative study. *Midwifery* 27 237–242. 10.1016/j.midw.2009.06.005 19783333

[B39] Shnaider P. Sijercic I. Wanklyn S. G. Suvak M. K. Monson C. M. (2017). The role of social support in cognitive-behavioral conjoint therapy for posttraumatic stress disorder. *Behav. Ther.* 48 285–294. 10.1016/j.beth.2016.05.003 28390493

[B40] Shulman S. (1993). Close relationships and coping behavior in adolescence. *J. Adolesc.* 16 267–283. 10.1006/jado.1993.1025 8282898

[B41] Slattery M. J. Grieve A. J. Ames M. E. Armstrong J. M. Essex M. J. (2013). Neurocognitive function and state cognitive stress appraisal predict cortisol reactivity to an acute psychosocial stressor in adolescents. *Psychoneuroendocrinology* 38 1318–1327. 10.1016/j.psyneuen.2012.11.017 23253895 PMC4077190

[B42] Smeds M. R. Janko M. R. Allen S. Amankwah K. Yoo P. (2019). Burnout and its relationship with perceived stress, self-efficacy, depression, social support, and programmatic factors in general surgery residents. *Am. J. Surg.* 219 907–912. 10.1016/j.amjsurg.2019.07.004 31307660

[B43] Suzuki M. Furihata R. Konno C. Kaneita Y. Uchiyama M. (2018). Stressful events and coping strategies associated with symptoms of depression: a Japanese general population survey. *J. Affect. Disord.* 238 482–488. 10.1016/j.jad.2018.06.024 29933216

[B44] Terry D. J. (1991). Coping resources and situational appraisals as predictors of coping behavior. *Pers. Individ. Differ.* 12 1031–1047. 10.1016/0191-8869(91)90033-8

